# Comparative Clinical Study Between Chlorhexidine Gel (0.2%) and Hyaluronic Gel (1%) in the Prevention of a Dry Socket After Tooth Extraction for Orthodontic Treatment

**DOI:** 10.7759/cureus.32391

**Published:** 2022-12-11

**Authors:** Abdulsalam Almushalbn, Ahmad Albassal, Munir Harfouch

**Affiliations:** 1 Department of Oral and Maxillofacial Surgery, Faculty of Dental Medicine, Damascus University, Damascus, SYR

**Keywords:** atraumatic extraction, dry socket, gelatin sponge, hyaluronic acid, chlorhexidine

## Abstract

Introduction

Several articles have investigated the intra-alveolar applications of bioadhesive gels containing various medicines and active chemicals, such as chlorhexidine (CHX) and hyaluronic acid (HA) to minimize the numerous postoperative sequelae, such as a dry socket. The purpose of this study was to investigate the postoperative outcomes of intra-alveolar application of 0.2% chlorhexidine or hyaluronic acid following atraumatic extraction.

Methods

A randomized clinical trial was conducted on a sample of 36 patients who need extraction of lower first premolar for orthodontic treatment. The patients were assigned randomly into two groups: CHX group and HA group. The signs and symptoms of the dry socket were evaluated on the third day in two groups.

Results

There was no statistically significant difference between the study and control groups when comparing the CHX group (P=0.2.6). In contrast, a significant difference was seen between the study and control sides in the HA group.

Conclusions

Within the limitation of this clinical trial, using hyaluronic gel with a gelatin sponge may be a preventive strategy for a dry socket following tooth extraction. After non-surgical tooth extraction, the topical administration of CHX with a gelatin sponge as a carrier did not seem to act as a protective substance against a dry socket.

## Introduction

Alveolar osteitis (AO) is a frequent inflammatory condition that can develop following tooth extraction. In addition to being known as a "dry socket," it causes symptoms that result in awful pain and require frequent hospital visits [1]. According to a recent definition, AO is "postoperative pain that increases between the first day and third day following the extraction and is accompanied by a dissolving blood clot in the alveolar socket, whether halitosis is present or not [2]. Clinical management of AO often involves alternating irrigation of the socket with 3% H_2_O_2 _and saline, curettage inducing fresh bleed under local anesthesia, and subsequent application of an iodoform strip. These extra procedures add to dentists' workloads and limit patients' ability to complete everyday tasks. Therefore, prevention is the best form of treatment [3,4]. Many researchers attempted to find effective methods to prevent AO, and several drugs have been proven to be effective. For example, chlorhexidine (CHX) is an antiseptic that is used frequently in therapeutic practice in the form of mouthwash and bioadhesive gel. According to previous studies, using 0.12% CHX mouthwash after surgery can reduce the risk of AO [5,6,7]. According to some studies, extraction sockets should be rinsed with CHX. Although the activity of CHX is brief, it is quick. Additionally, its regular use as an oral rinse can have negative consequences such as dysgeusia, oral mucosa lesions, external dental discoloration, and dental calculus formation. Therefore, to avoid AO, dentists or researchers are thinking about using CHX gel rather than CHX mouthwash [8,9].

Recent spectroscopy experiments have verified that hyaluronic acid (HA) is capable of neutralizing reactive oxygen species to lessen the toxicity of free radicals, which is in line with the substance's antioxidant characteristics [10]. HA primarily exhibits bacteriostatic properties at high concentrations (0.8%) [9]. Recent years have seen the publication of various scientific studies describing the use of HA as an intra-alveolar bioadhesive gel to minimize extraction-related postoperative problems [11,12].

To date, no clinical trial has been conducted to compare the possible beneficial effects of CHX and HA after atraumatic extraction. The aim of this study was to examine the postoperative effects of intra-alveolar placement of 0.2% CHX or 1% HA bioadhesive gels following atraumatic extraction.

## Materials and methods

Study design and registration

A randomized comparative clinical investigation using the split mouth approach was carried out in the outpatient clinics of the Oral and Maxillofacial Department - Faculty of Dentistry - Damascus University between June 2021 and February 2022. The Damascus University Research Ethics Committee approved this clinical study (registration no. 2021-1021). After explaining the study's goals and methodology to the patients verbally and in writing, they gave their signed consent to participate in the study.

Patients’ recruitment and eligibility criteria

The research sample consisted of 72 sockets of mandibular first premolar from 36 patients who met the inclusion criteria. The inclusion criteria were as follows: (1) patients' age ranged between 18 and 35 years, (2) any patient who is classified as ASA1 (a patient without any systemic disease) or ASA2 who requires the extraction of symmetrical single root teeth in the lower jaw (a patient with a mild systemic disease that does not affect their general health), (3) the patients' oral health is good (they don't have gingivitis or periodontitis) and neither local anesthetic nor minimally invasive oral surgery is contraindicated, and (4) the patient's commitment to attend follow-up.

Exclusion criteria were as follows: (1) patients who have syndromes or developmental anomalies were excluded from the study, (2) disorders of the periodontal tissues that are advanced and uncontrolled, (3) patients who are smokers and alcoholics or those using immunosuppressants and corticosteroids, (4) pregnant or nursing women, (5) patients who have cysts or tumors at the treatment site, and (6) individuals who underwent radiation for head and neck cancers.

Using the web tool www.random.org, the patients were randomly divided into two groups (n = 18 for each group).

In group 1 (CHX group), there were 36 lower first premolars sockets from 18 patients. A 10ml gel of 0.2% CHX (PerioKin, Kin, Barcelona, Spain) was applied to a gelatin sponge (GelSpon® “Bovine Origin”, Eucare Pharmaceuticals, Chennai, India), which could be used as a carrier. Nylon sutures 4-0 (Anhui Kangning Industrial (Group) Co., LTD, Tianchang, Anhui, China) were used in an X shape to fix the dressing. On the opposite side, a gelatin sponge dressing was used, and 4-0 nylon sutures were placed in an X pattern.

In group 2 (HA group), there were 36 lower first premolar sockets from 18 patients who made up this group. A 10 ml gel of HA 1% concentration (RICEFARMA S.R.L, Milano, Italy) was applied to a gelatin sponge dressing to fill the socket with the same suture material. Gelatin sponge dressing was placed and 4-0 nylon sutures on the opposite side.

Surgical procedure and outcome measures

Under local anesthesia (lidocaine 2% with adrenaline 1/80.000), a sulcular incision is preferably made 360 degrees around the tooth with a periotome. This was done with an intention to secure all fibers of connective tissue attachment above the level of the bone are cut. If these fibers are not cut before extraction, they will cause more tissue damage and possibly even fracture the buccal bone plate.

The tooth is then luxated using a proximator (SA-JK Surgical Corporation, Sialkot, Pakistan); the long axis of the proximator blade is inserted into the ligamentous area along the longitudinal axis of the root to protect the vestibular plate of bone, and it is paused for 10 to 30 seconds while the instrument is in place to allow expanding the alveolar bone. The proximator is used as a lever to apply pressure to the tooth root after first being employed as a moveable wedge. The dental forceps are used at the end to hold the tooth, move it buccally and lingually, and rotate it when a significant movement of the teeth is achieved. After the tooth is pulled, 0.2% CHX (group 1) and 1% HA (group 2) were mixed with a gelatin carrier and inserted into the alveoli. Finally, a 4-0 non-resorbable suture was used to close the wounds. Paracetamol 0.5 g every eight hours for three days was the only postoperative medication used in every case if there was pain.

**Figure 1:**
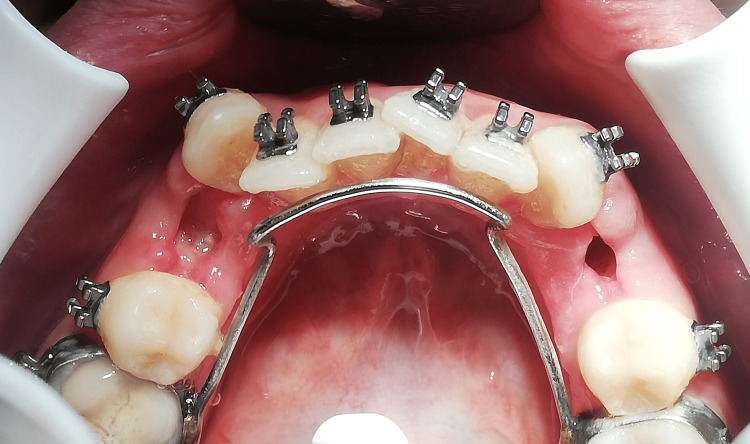
Clinical assessment of the alveolar socket on the third day Right-sided normal healing of the alveolar socket; left-sided inflammatory healing of the alveolar socket (alveolar osteitis).

The clinical assessments of the dry socket were performed by observing the signs and symptoms of a dry socket, which include exposed bone, pain, and halitosis on the third day after the extraction (Figure 1).

Statistical analysis

Data were gathered, and they were shown as frequencies and percentages. Chi-square tests were used in statistical studies with IBM SPSS Statistics for Windows, Version 19 (Released 2010; IBM Corp., Armonk, New York, United States). The statistical significance was P<0.05.

## Results

None of the patients who were invited to participate in the experiment and met the inclusion criteria were declined. The mean patient age was 21.9 years in the CHX group and 23.1 in the HA group. Sample characteristics are presented by comparing the two study groups which are presented in Table 1. Intra-group comparisons of the CHX group showed no significant differences between the study and control groups (P=0.206) and are shown in Table 2, and intra-group comparisons of the HA group showed significant differences between the study and control groups (P=0.016) and are shown in Table 3.

**Table 1:** Sample characteristics (age and sex). n: number of patients; SD: standard deviation

Variable	Chlorhexidine group (n=18)	Hyaluronic acid group (n=18)
Age, mean±SD	21.9±4.3	23.1±4.9
Sex, n (%)		
Male	7 (38.9)	7 (38.9)
Female	11 (61.1)	11 (61.1)

 

**Table 2:** Comparisons of the frequencies and percentages of the inflammatory response in the chlorhexidine group.

Chlorhexidine group	Frequencies			Percentages			P-value
	There is inflammation	No inflammation	Total	There is inflammation	No inflammation	Total	
Study group	2	16	18	11.1%	88.9%	100%	0.206
Control group	5	13	18	27.8%	72.2%	100%	

 

**Table 3:** Comparisons of the frequencies and percentages of the inflammatory response in the hyaluronic acid group.

Hyaluronic acid group	Frequencies			Percentages			P-value
	There is inflammation	No inflammation	Total	There is inflammation	No inflammation	Total	
Study group	0	18	18	0%	100%	100%	0.016
Control group	5	13	18	27.8%	72.2%	100%	

## Discussion

In general practice, tooth extraction is one of the most often performed procedures. The common extraction techniques include luxation using an elevator, severing the periodontal connection, and removal with forceps. If endodontic therapy has already compromised the tooth or roots are long or/and dilacerated, traditional extraction forceps frequently fracture the tooth, the surrounding bone, or both. In addition to requiring a more involved surgical procedure, this could lead to unfavorable postoperative complications [13]. Traumatic extractions, in which strong luxation or forceps forces are needed to extract teeth, increase the risk of developing dry socket lesions, which affect one to five percent of all extractions [14].

Numerous studies have looked into the intra-alveolar applications of bioadhesive gels with different pharmaceuticals and active chemicals, such as CHX [15,8] and HA [11,12], to reduce the various postoperative complications, such as a dry socket in addition to conventional drug treatment. However, no trials have yet been conducted to examine the potential benefits of CHX and HA following an atraumatic extraction. Because of this, the effects of inserting 1% HA or 0.2% CHX bioadhesive gels into the alveoli after atraumatic extraction were tested in this clinical trial. We chose not to employ traditional extraction techniques since they could induce gingival tissue laceration and even result in the loss of the buccal bony plate and interdental bone crest. Trismus, a dry socket, and postoperative discomfort are other complications [16].

In the course of a traumatic extraction, strong luxation or forceps forces are transferred to the jawbone that surrounds the roots which may cause the bone to be crushed on the intaglio surface of the extraction socket. The osteoblasts in the extraction socket may die or undergo apoptosis as a result [14]. One of the dental issues that have received the most attention is dry sockets, and numerous studies have been conducted to find a successful approach to managing and preventing it [17]. One of the most popular mouthwashes is CHX mouthwash, which has the reputation of lowering the risk of AO. However, Hita Iglesias et al. found that CHX gel has more promising results than mouthwash [18].

The longer exposure time and slower medication release in the early postoperative period, as well as the fact that patients' cooperation is not required, are crucial when using mouthwash. The antibacterial properties of this agent may also be to blame for the lower frequency of AO. Byproducts of bacterial infection are said to increase antifibrinolytic activity, which causes clot disintegration and loss that cause AO. According to published research, the AO is prevented by CHX gels that inhibit fibrinolytic activity [18].

Muñoz-Cámara et al. found that 1% HA (0%), 0.2% CHX (6.67%), and control (10%) represented the lowest to the highest percentage of instances of AO [10]. The bactericide action of 1% HA, which lowers the intra-alveolar bacterial load and has regenerating potential, may be the cause of the preventing effect on AO (measured by hyaladherins that promote cell proliferation, migration, and angiogenesis mechanisms, so facilitating rapid tissue regeneration).

Rubio-Palau et al. conducted a double-blind randomized clinical trial with 160 patients (80 receiving 0.2% CHX bioadhesive gel and 80 receiving placebo) and discovered a statistically significant difference in the incidence of AO between the study group (17.5%) and the control group, with the incidence of the condition being higher in the control group (22.5%) [19]. Sridhar et al. conducted a split-mouth clinical trial and observed that AO was recorded in 8% of the control group after extractions of bilateral impacted mandibular third molars, while it was not present at extraction sites treated with intra-alveolar applications of 0.2% CHX bioadhesive gel, indicating a statistically significant difference between the groups (P = 0.041) [20]. We disagree with two previous studies [19,20], which may be attributed to the difference in surgical extraction and the region of extraction.

HA can form a healthy coagulum for a longer amount of time due to its adhesive capability, which is one of the benefits of local application [21]. Comparing the use of Alvogyl® alone to the use of HA, it is shown that the latter reduces the number of alveolar osteitis symptoms and signs and speeds up the removal of painful feelings [22]. HA demonstrated benefits including analgesic, reparative, and regenerative properties, high elasticity, biocompatibility, biodegradability, and low immunogenicity which led to the suggestion that it might be used to treat AO [23].

We agree with a previous randomized controlled trial that used 0.2% bioadhesive CHX gel after surgically extracting the mandibular third molar reducing the risk of developing AO by 2.3 times. Therefore, this gel should be used prophylactically to avoid complications and decrease the financial burden on patients [17].To the best of our knowledge, no previous clinical studies have examined the potential positive effect of intra-alveolar application of CHX and HA after atraumatic extraction.

There were minimal methodological limitations to this study, which include: (1) the study did not include an extraction socket of the upper jaw, (2) there was no histological study on experimental animals, and (3) no radiographic studies were performed to investigate bone healing.

## Conclusions

Within the limitation of this clinical study, applying hyaluronic gel with a gelatin sponge may be a preventive measure for a dry socket after tooth extraction. The topical application of CHX with a gelatin sponge as a carrier did not appear as a protective material from a dry socket after non-surgical tooth extraction. We also found that an absorbable gelatin sponge is an acceptable carrier to deliver CHX and hyaluronic gel.

## References

[1] Kolokythas A, Olech E, Miloro M (2010). Alveolar osteitis: a comprehensive review of concepts and controversies. Int J Dent.

[2] Blum IR (2002). Contemporary views on dry socket (alveolar osteitis): a clinical appraisal of standardization, aetiopathogenesis and management: a critical review. Int J Oral Maxillofac Surg.

[3] Ishihama K, Kimura T, Yasui Y, Komaki M, Ota Y (2006). Azithromycin as prophylaxis for the prevention of postoperative infection in impacted mandibular third-molar surgery. J Infect Chemother.

[4] Wiśniewska I, Slósarczyk A, Myśliwiec L, Sporniak-Tutak K (2009). [Lincomycin applied to the alveolus on TCP carrier and its effect on wound healing after surgical extraction of a third molar]. Ann Acad Med Stetin.

[5] Caso A, Hung LK, Beirne OR (2005). Prevention of alveolar osteitis with chlorhexidine: a meta-analytic review. Oral Surg Oral Med Oral Pathol Oral Radiol Endod.

[6] Field EA, Nind D, Varga E, Martin MV (1988). The effect of chlorhexidine irrigation on the incidence of dry socket: a pilot study. Br J Oral Maxillofac Surg.

[7] Hermesch CB, Hilton TJ, Biesbrock AR, Baker RA, Cain-Hamlin J, McClanahan SF, Gerlach RW (1998). Perioperative use of 0.12% chlorhexidine gluconate for the prevention of alveolar osteitis: efficacy and risk factor analysis. Oral Surg Oral Med Oral Pathol Oral Radiol Endod.

[8] Rodríguez-Pérez M, Bravo-Pérez M, Sánchez-López JD, Muñoz-Soto E, Romero-Olid MN, Baca-García P (2013). Effectiveness of 1% versus 0.2% chlorhexidine gels in reducing alveolar osteitis from mandibular third molar surgery: a randomized, double-blind clinical trial. Med Oral Patol Oral Cir Bucal.

[9] Torres-Lagares D, Gutierrez-Perez JL, Infante-Cossio P, Garcia-Calderon M, Romero-Ruiz MM, Serrera-Figallo MA (2006). Randomized, double-blind study on effectiveness of intra-alveolar chlorhexidine gel in reducing the incidence of alveolar osteitis in mandibular third molar surgery. Int J Oral Maxillofac Surg.

[10] Muñoz-Cámara D, Pardo-Zamora G, Camacho-Alonso F (2021). Postoperative effects of intra-alveolar application of 0.2% chlorhexidine or 1% hyaluronic acid bioadhesive gels after mandibular third molar extraction: a double-blind randomized controlled clinical trial. Clin Oral Investig.

[11] Gocmen G, Aktop S, Tüzüner B, Goker B, Yarat A (2017). Effects of hyaluronic acid on bleeding following third molar extraction. J Appl Oral Sci.

[12] Guazzo R, Perissinotto E, Mazzoleni S, Ricci S, Peñarrocha-Oltra D, Sivolella S (2018). Effect on wound healing of a topical gel containing amino acid and sodium hyaluronate applied to the alveolar socket after mandibular third molar extraction: a double-blind randomized controlled trial. Quintessence Int.

[13] Kapila S, Kaur T, Bhullar RS, Sandhu A, Dhawan A, Kaur A (2020). Use of physics forceps in atraumatic orthodontic extractions of bilateral premolars: a randomized control clinical study. J Maxillofac Oral Surg.

[14] Mamoun J (2018). Dry socket etiology, diagnosis, and clinical treatment techniques. J Korean Assoc Oral Maxillofac Surg.

[15] Madrazo-Jiménez M, Rodríguez-Caballero Á, Serrera-Figallo MÁ, Garrido-Serrano R, Gutiérrez-Corrales A, Gutiérrez-Pérez JL, Torres-Lagares D (2016). The effects of a topical gel containing chitosan, 0,2% chlorhexidine, allantoin and despanthenol on the wound healing process subsequent to impacted lower third molar extraction. Med Oral Patol Oral Cir Bucal.

[16] Madathanapalli S, Surana S, Thakur D, Ramnani P, Kapse S (2016). Physics forceps vs conventional forceps in extraction of maxillary first molar. Int J Oral Care Res.

[17] Shad S, Hussain SM, Tahir MW, Rahat Geelani SR, Khan SM, Abbasi MM (2018). Role of 0.2% bio-adhesive chlorhexidine gel in reducing incidence of alveolar osteitis. J Ayub Med Coll Abbottabad.

[18] Hita-Iglesias P, Torres-Lagares D, Flores-Ruiz R, Magallanes-Abad N, Basallote-Gonzalez M, Gutierrez-Perez JL (2008). Effectiveness of chlorhexidine gel versus chlorhexidine rinse in reducing alveolar osteitis in mandibular third molar surgery. J Oral Maxillofac Surg.

[19] Rubio-Palau J, Garcia-Linares J, Hueto-Madrid JA, González-Lagunas J, Raspall-Martin G, Mareque-Bueno J (2015). Effect of intra-alveolar placement of 0.2% chlorhexidine bioadhesive gel on the incidence of alveolar osteitis following the extraction of mandibular third molars. A double-blind randomized clinical trial. Med Oral Patol Oral Cir Bucal.

[20] Sridhar V, Wali GG, Shyla HN (2011). Evaluation of the perioperative use of 0.2% chlorhexidine gluconate for the prevention of alveolar osteitis after the extraction of impacted mandibular third molars: a clinical study. J Maxillofac Oral Surg.

[21] Rabasseda X (2000). The role of hyaluronic acid in the management of periodontal disease. Drugs of Today.

[22] Dubovina D, Mihailović B, Bukumirić Z, Vlahović Z, Miladinović M, Miković N, Lazić Z (2016). The use of hyaluronic and aminocaproic acid in the treatment of alveolar osteitis. Vojnosanit Pregl.

[23] King SR, Hickerson WL, Proctor KG (1991). Beneficial actions of exogenous hyaluronic acid on wound healing. Surgery.

